# Differences in epidemiology of enteropathogens in children pre- and post-rotavirus vaccine introduction in Kilifi, coastal Kenya

**DOI:** 10.1186/s13099-022-00506-z

**Published:** 2022-08-01

**Authors:** Charles N. Agoti, Martin D. Curran, Nickson Murunga, Moses Ngari, Esther Muthumbi, Arnold W. Lambisia, Simon D. W. Frost, Barbara A. Blacklaws, D. James Nokes, Lydia N. Drumright

**Affiliations:** 1 Epidemiology and Demography Department, Kenya Medical Research Institute (KEMRI)-Wellcome Trust Research Programme, P.O. Box 230, Kilifi, 80108 Kenya; 2 School of Health and Human Sciences, Pwani University, Kilifi, Kenya; 3 Public Health England, Cambridge, UK; 4 Clinical Microbiology and Public Health Laboratory, Addenbrooke’s Hospital, Cambridge, UK; 5 Microsoft Research, Building 99, 14820 NE 36th St., Redmond, WA 98052 USA; 6 Department of Infectious Disease Epidemiology, Faculty of Epidemiology and Public Health, London School of Hygiene and Tropical Medicine, Keppel St, Bloomsbury, London, WC1E 7HT UK; 7 Department of Veterinary Medicine, University of Cambridge, Cambridge, UK; 8 School of Life Sciences, University of Warwick, Coventry, UK; 9 Department of Medicine, University of Cambridge, Cambridge, UK; 10 Department of Medicine, University of Washington, Washington, USA

**Keywords:** Enteropathogens, RVA, Taqman array card, Co-infections, Vaccination, Children, Epidemiology, Kenya

## Abstract

**Background:**

Kenya introduced Rotarix^®^ (GlaxoSmithKline Biologicals, Rixensart, Belgium) vaccination into its national immunization programme beginning July 2014. The impact of this vaccination program on the local epidemiology of various known enteropathogens is not fully understood.

**Methods:**

We used a custom TaqMan Array Card (TAC) to screen for 28 different enteropathogens in 718 stools from children aged less than 13 years admitted to Kilifi County Hospital, coastal Kenya, following presentation with diarrhea in 2013 (before vaccine introduction) and in 2016–2018 (after vaccine introduction). Pathogen positivity rate differences between pre- and post-Rotarix^®^ vaccination introduction were examined using both univariate and multivariable logistic regression models.

**Results:**

In 665 specimens (92.6%), one or more enteropathogen was detected, while in 323 specimens (48.6%) three or more enteropathogens were detected. The top six detected enteropathogens were: enteroaggregative *Escherichia coli* (EAggEC; 42.1%), enteropathogenic *Escherichia coli* (EPEC; 30.2%), enterovirus (26.9%), rotavirus group A (RVA; 24.8%), parechovirus (16.6%) and norovirus GI/GII (14.4%). Post-rotavirus vaccine introduction, there was a significant increase in the proportion of samples testing positive for EAggEC (35.7% vs. 45.3%, *p = 0.014*), cytomegalovirus (4.2% vs. 9.9%, *p = 0.008*), *Vibrio cholerae* (0.0% vs. 2.3%, *p = 0.019*), *Strongyloides* species (0.8% vs. 3.6%, *p = 0.048*) and *Dientamoeba fragilis* (2.1% vs. 7.8%, *p = 0.004*). Although not reaching statistical significance, the positivity rate of adenovirus 40/41 (5.8% vs. 7.3%, *p = 0.444*), norovirus GI/GII (11.2% vs. 15.9%, *p = 0.089*), *Shigella* species (8.7% vs. 13.0%, *p = 0.092*) and *Cryptosporidium* spp. (11.6% vs. 14.7%, *p = 0.261*) appeared to increase post-vaccine introduction. Conversely, the positivity rate of sapovirus decreased significantly post-vaccine introduction (7.8% vs. 4.0%, *p = 0.030*) while that of RVA appeared not to change (27.4% vs. 23.5%, *p = 0.253*). More enteropathogen coinfections were detected per child post-vaccine introduction compared to before (mean: 2.7 vs. 2.3; *p = 0.0025*).

**Conclusions:**

In this rural Coastal Kenya setting, childhood enteropathogen infection burden was high both pre- and post-rotavirus vaccination introduction. Children who had diarrheal admissions post-vaccination showed an increase in coinfections and changes in specific enteropathogen positivity rates. This study highlights the utility of multipathogen detection platforms such as TAC in understanding etiology of childhood acute gastroenteritis in resource-limited regions.

**Supplementary Information:**

The online version contains supplementary material available at 10.1186/s13099-022-00506-z.

## Introduction

In 2016, there were approximately 446,000 deaths worldwide caused by diarrheal illnesses among children aged < 5 years, with the majority occurring in low-income countries [1, 2]. Rotavirus group A (RVA) was estimated to be responsible for approximately 128,000 of these deaths.

The World Health Organization (WHO) recommended inclusion of RVA vaccines into national immunization programs (NIPs) of all countries in 2009 [3]. As of November 2021, 114 countries had included rotavirus vaccination into their NIPs [4]. Kenya included Rotarix^®^ (GlaxoSmithKline Biologicals, Rixensart, Belgium), one of the WHO pre-qualified rotavirus vaccines, in its NIP in July 2014 [3], with doses given at 6 and 10 weeks of age. Based on antigen testing (enzyme immunoassay; EIA), Kilifi County Hospital (KCH), Coastal Kenya, reported a 57% reduction in rotavirus hospitalizations in the first year after vaccine introduction and an 80% reduction in the second year among children < 5-year-olds [4]. Further, KCH found a 64% Rotarix^®^ vaccine effectiveness in children aged < 5 years [5], which is similar to other low-income settings in sub-Saharan Africa [5, 6], but lower than high-income settings [7, 8].

Over 30 potential enteropathogens can cause diarrhea. In addition to a reduction in RVA-induced diarrhea, a referral peri-urban hospital in Central Kenya observed a 31% decrease in all-cause diarrheal hospital admissions in the first year and a 58% decrease in the second year [9]. While RVA has clearly been responsible for a large burden of severe diarrheal cases among children < 5 years-old, there are many other enteropathogens that contribute to severe diarrheal illness in Kenya [10–13] but their epidemiology, both pre- and post-rotavirus vaccine introduction, is under-studied. Previously, we examined change in prevalence of 5 enteric viruses, including RVA, pre- and post-rotavirus vaccination demonstrating an increase in norovirus GII and a decrease in RVA, however this study did not examine many other potential infectious causes of diarrheal illness [14].

Using a custom TaqMan Array Card (TAC), this study aimed to compare the epidemiological patterns of 28 different enteropathogens in children < 13 years-old admitted to KCH with diarrhea, pre- and post-rotavirus vaccine introduction.

## Methods

### Study design and population

KCH is a referral facility that primarily serves residents of Kilifi County located on the Kenyan Coast [15]. From September 2009, a surveillance study of rotavirus was established at KCH pediatric ward targeting children < 13 years old who presented with diarrhea (defined as passing three or more loose stools in a 24-h period with or without visible blood) [16]. At KCH, pediatric admission is determined by the clinician’s assessment of the severity of the illness and children must stay overnight once admitted. A single stool specimen was collected within 48 h of admission and immediately put into − 4 °C before transferring to the adjoining KEMRI-Wellcome Trust Research Programme (KWTRP) laboratories for long-term storage at −  80 °C. The current retrospective analysis targeted samples collected in the year 2013 (pre-vaccine period) and 2016–2018 (post-vaccine period). During these periods, sample collection was interrupted 6 times by healthcare worker strikes; 3 times each pre- and post-vaccine introduction [17].

### Sample processing

Total nucleic acid (TNA) was extracted from stool using two different approaches. First, the *cador* Pathogen 96 QIAcube HT Kit (QIAGEN, Hilden, Germany) with 200 µL liquid or 200 mg solid stool was used on samples from 2013. Second, the QIAamp Fast DNA Stool Mini Kit (QIAGEN, Hilden, Germany) with 200 µL or 200 mg stool after bead-beating and centrifugation to remove debris was used on samples from 2016 to 2018. An extraction blank was included in every processing batch for quality control. TNA extracts were stored at − 80 °C for up to 130 weeks and defrosted once, only for the enteropathogen diagnostics.

### TaqMan array card (TAC) analysis

**Fig. 1:**
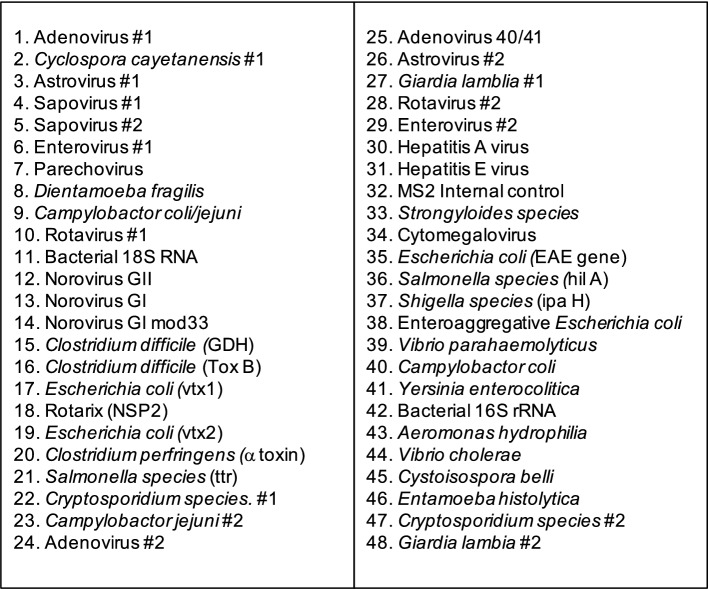
The configuration of the GASTRO v4.0 K TaqMan Array Card used for screening 28 enteropathogens. Some of the enteropathogens had multiple spots (targets) on the card

A custom TAC spotted with lyophilized target-specific primers and probes was used to detect enteropathogens from the TNA extracts. The GASTRO v4.0K TAC was designed by MC at Public Health England (PHE), Cambridge, England [18] and manufactured by ThermoFisher, USA. GASTRO v4.0K screened for 28 enteropathogens: 12 viruses, 10 bacteria, 5 protozoa and 1 helminth (Fig. 1 and Additional file 1: Table S1). All TAC targets were validated previously by PHE and include 16S bacterial and 18S RNA as internal controls, and bacteriophage MS2 as an external control. A Rotarix^®^ vaccine-specific assay targeting the NSP2 gene was included to identify RVA vaccine shedding due to recent vaccination. The molecular probes were labeled at the 5’ end with 6-carboxyfluorescein (FAM) reporter dye and NFQ-MGB quencher dye at the 3’ end [18].

For each TAC, 8 samples were tested (48-wells per sample; Fig. 1). A mix of 60 µL of nuclease-free water, 25 µL of TaqMan Fast Virus 1-Step MasterMix (ThermoFisher, USA) containing ROX reference dye and 15 µL of TNA was prepared and loaded onto the TAC sample portal. TACs were spun twice at 1200 rpm for two minutes and sealed. TACs were processed using the Quantistudio-7-Flex Real-time PCR System (Thermofisher, USA). The thermocycling conditions were 50 °C for 5 min followed by 45 cycles of 95 °C for 1 s and 60 °C for 20 s. Extraction blanks and no template controls were included once in every 10 runs for quality control.

Amplification data were analyzed by the cycle threshold (Ct) method (QuantiStudio™Real-Time PCR software v.1.1). A threshold value of 0.2 florescence units was applied to all our analyses and baseline range set to automatic. The amplification curves for presumptive positive samples were all visually inspected for confirmation. Pathogen prevalence was evaluated at Ct cut-off levels; <40.0, < 35.0 and < 30.0.

### Clinical and vaccination data

Clinical data were collected using a standard clinical research form (CRF) entered into an electronic database [19]. The CRF included admission date, discharge date, presenting signs and symptoms, co-morbidities, and discharge diagnosis and outcome. Diarrhea was categorized as acute (< 7 days), prolonged (7–13 days), persistent (14–28 days), and chronic (> 28 days). Diarrheal severity was assessed by Vesikari Clinical Severity Score (VCSS) [20]. All samples were tested for RVA using a commercial EIA antigen detection kit (ProsPeCT, Oxoid, Thermo Scientific). All pediatric admissions were offered HIV testing using two rapid antibody tests according to national guidelines [21].

Rotavirus vaccination status for children was obtained from the Kilifi electronic vaccine registry [22]. The registry captures the child’s vaccination data real-time in clinics within the Kilifi health and demographic surveillance system (KHDSS) operated by KWTRP. This is then linked with KCH surveillance data using unique KHDSS identification numbers.

### Analyses

We compared clinical and demographic characteristics of (i) included and excluded children and (ii) those recruited pre- and post-rotavirus vaccination using chi-squared analysis and Fisher’s exact test for categorical variables and Wilcoxon-rank sum and t-tests for continuous variables. As this was the first use of this TAC in Kenya, we descriptively explored different lower limits of detection (Ct ≤ 30, 35, 40) for a positive result. Using Ct ≤ 35 as the lower limit of detection, pathogen prevalence was calculated as the proportion positive over the total tested with 95% confidence intervals (CI) calculated using the exact method. We assessed differences in pathogen presence in pre-post-rotavirus vaccination periods by univariate logistic regression. Any enteropathogen that was marginally different (*p* < 0.1) in univariate analyses, as well as rotavirus (due to epidemiological interest), were included in multivariable logistic regression analyses adjusted for age, sex, Vesikari score, HIV-status, hospital length of stay (LoS), and fatality. Separate multivariable logistic regression analyses per pathogen were conducted due to the high prevalence of co-infections. The analyses were performed in Stata version 16.1 and R version 3.6.3.

### Ethical considerations

Before samplecollection, informed written consent was gained from each child’s parent/guardian. The Scientific and Ethics Review Unit (SERU) at Kenya Medical Research Institute, Nairobi, approved the study protocol (Protocol #3624).

## Results

### Participant characteristics

A total of 1317 KCH pediatric admissions with diarrhea were eligible for inclusion during study periods (2013, 2016–2018). Of these, 1250 (95%) were unique patients; 718 (57.4%) provided an adequate stool sample for TAC processing. Children who did and did not have a stool sample were similar except those with a stool sample: (i) were more likely to have reported vomiting at admission (*p = 0.001*); (ii) were hospitalized for longer (mean 5.8 days versus 4.3 days, *p < 0.001*); (iii) were more likely to have been discharged alive (*p < 0.001*); (iv) were more likely to have or have been exposed to HIV (*p = 0.049*); and (v) were less likely to be included in 2017 and 2018 than in 2013 and 2016 (*p < 0.001*) (Table 1).

**Table 1:** Demographic and clinical characteristics of the eligible, included and excluded participants and, among participants, pre-vaccine versus the post-vaccine introduction period comparisons

Characteristic	All eligible (n = 1250) n (%)	Included (n = 718) n (%)	Excluded* (n = 532) n (%)	Included vs Excluded p-value	Pre-vaccine (n = 241) n (%)	Post-vaccine (n = 477) n (%)	Pre- vs Post-Vaccine p-value
Sex (Male)	697 (55.8)	402 (56.0)	295 (55.5)	0.0850	139 (57.7)	263 (55.1)	0.517
Median age (IQR) *in months*	13.8 (8.2–24.6)	13.3 (8.0–23.0)	14.3 (8.5–26.6)	0.081	11.5 (6.8–21.5)	14.1 (8.5–24.2)	**0.004**
Age category (in months)				0.115			**0.050**
0–11	528 (42.2)	323 (45.0)	205 (38.5)		126 (52.3)	197 (41.3)	
12–23	402 (32.2)	224 (31.2)	178 (33.5)		65 (27.0)	159 (33.3)	
24–59	203 (16.2)	106 (14.8)	97 (18.2)		31 (12.9)	75 (15.7)	
> 60	117 (9.4)	65 (9.1)	52 (9.8)		19 (7.9)	46 (9.6)	
Admission year				** < 0.001**			
Year 2013	351 (28.1)	241 (33.6)	110 (20.7)		241 (100.0)	-	NA
Year 2016	348 (27.8)	245 (34.1)	103 (19.4)		-	252 (51.4)	
Year 2017	196 (15.7)	61 (8.5)	135 (25.4)		-	641(12.8)	
Year 2018	355 (28.4)	171 (23.8)	184 (34.6)		-	171 (35.9)	
HIV status				**0.049**			** < 0.001**
Negative	994 (79.5)	565 (78.7)	429 (80.6)		171 (71.0)	394 (82.6)	
Infected/exposed	78 (6.24)	55 (7.7)	23 (4.3)		14 (5.8)	41 (8.6)	
Unknown	178 (14.2)	98 (13.7)	80 (15.0)		56 (23.2)	42 (8.8)	
Diarrhea history at admission				0.497			0.371
Acute	1130 (90.4)	657 (91.5)	473 (88.9)		222 (92.1)	435 (91.2)	
Prolonged	79 (6.3)	40 (5.6)	39 (7.3)		10 (4.2)	30 (6.3)	
Persistent	10 (0.8)	5 (0.7)	5 (0.9)		3 (1.2)	2 (0.4)	
Chronic	31 (2.5)	16 (2.2)	16 (2.2)		6 (2.5)	10 (2.1)	
Bloody diarrhea (n = 1245)	75 (6.0)	42 (5.9)	33 (6.2)	0.796	17 (7.1)	25 (5.3)	0.328
Vomiting at admission	817 (65.4)	498 (69.4)	319 (60.0)	**0.001**	171 (71.0)	327(68.6)	0.510
Hospital length of stay							
Mean (SD#)	5.2 (5.0)	5.8 (5.4)	4.3 (4.3)	** < 0.001**	5.5 (5.5)	5.9 (5.3)	0.317
Median (IQR)	4 (2–6)	4 (3–7)	3 (1–6)	** < 0.001**	4 (2–7)	5 (3–7)	**0.005**
Vesikari Clinical Severity Score							
Mean (SD#)	11.4 (2.3)	11.5 (2.3)	11.3 (2.3)	0.183	11.7 (2.5)	11.4 (2.1)	0.062
Median (IQR)	11 (10–13)	11 (10–13)	11 (10–13)	0.182	11 (10–13)	11 (10–13)	0.289
Died before discharge	145 (11.6)	39 (5.4)	106 (19.9)	** < 0.001**	8 (3.2)	31 (6.5)	0.076

^*^ Due to insufficient stool sample provision; the bold emphasis of some of the* p* -values indicate those statistically significant

The children who had provided sufficient stool had a median age of 13.3 months (IQR: 8.0–23.0). Clinical and demographic characteristics of children from pre- and post-vaccine introduction periods (n = 718) were similar except that those from the pre-vaccine period were (i) significantly younger (11.5 vs. 14.1 months, *p = 0.004*); (ii) less likely to have known HIV exposure, but more likely to have unknown HIV status (*p < 0.001*); and (iii) had a lower median hospital stay (4 vs. 5 days, *p = 0.005*) than those from the post-vaccine period (Table 1).

### Pathogen positivity rate across different ct cut-offs

**Fig. 2:**
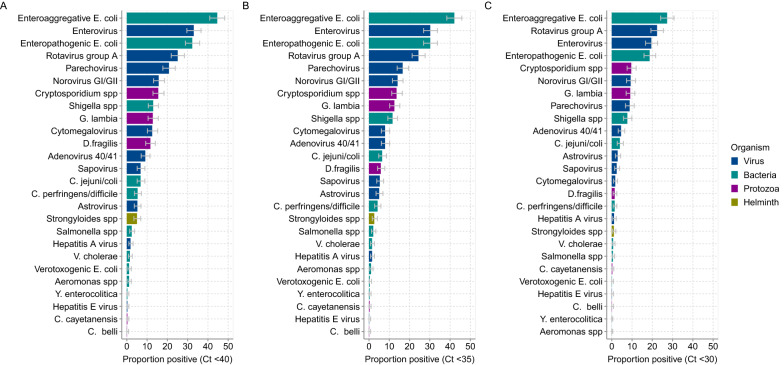
Prevalence of enteropathogens in stool samples from 718 children hospitalised at KCH at different PCR cycle threshold (Ct) cut-offs. The bars represent organism type and include 95% confidence interval errors bars for the proportions. Panel (**a**) prevalence of the detected enteropathogens when applying a Ct cut-off of ≤ 40 to define positives. Panel (**b**) prevalence of the detected enteropathogens when applying a Ct cut-off ≤ 35 to define positives. Panel (**c**) prevalence of detected the enteropathogens when applying a Ct cut-off ≤ 30 to define positives

Of the 718 specimens analyzed by TAC, using a cycle threshold cut-off of ≤ 40.0 Ct (which was considered an appropriate cut-off based on clinical samples from the UK [18, 23]) one or more enteropathogen was detected in 94.0% of the children (n = 675). Overall, the top five enteropathogens detected at the ≤ 40.0 Ct cut off value were: enteroaggregative *Escherichia coli* (EAggEC) 44.6%; enterovirus 33.0%; enteropathogenic *Escherichia coli* (EPEC) 32.3%; RVA 25.1%; and parechovirus 20.8% (Fig. 2A). When the limit of detection was lowered to ≤ 30 Ct (high pathogen burden), 12/26 of detected pathogens decreased in prevalence by a factor of 2 or more (Fig. 2B). The largest declines were observed with verotoxogenic *E. coli* (VTEC) (11 fold), *Dientamoeba fragilis* (8.3 fold), cytomegalovirus (7.4 fold), *Strongyloides* spp. (5.0 fold), *Salmonella* spp. (4.0 fold), *Clostridium perfringens/difficile* (3.8 fold) and hepatitis E virus (3.0 fold). Only three targets (*D. fragilis*, hepatitis E virus, and VTEC) showed a reduction by factor 2 or more when comparing a Ct value of ≤ 40 versus ≤ 35 as the limit of detection. On average, the prevalence of the detected pathogens decreased by 1.3 fold when the Ct cut-off was lowered from 40.0 to 35.0 (Fig. 2C). Notably, even with the most stringent Ct cut-off (≤ 30), the prevalence of RVA was unaffected: 25.1% (21.9–28.4) at Ct ≤ 40 versus 22.3% (19.3–25.5) at Ct ≤ 30. No samples tested positive for *Vibrio parahaemolyticus, Entamoeba histolytica* or the Rotarix® vaccine strain at any Ct detection limit. All subsequent analyses have used the Ct cut-off of ≤ 35.0 for all targets. Detection above this threshold has previously been considered clinically insignificant or irreproducible in studies from low-income settings [24, 25].

### Pathogen positivity rate pre-post-rotavirus vaccine introduction

Among the 718 samples, 241 (33.6%) were from the pre-vaccine period (2013) and 477 (66.4%) were from the post-vaccine introduction period (2016–2018; Table 2). In univariate logistic regression analyses a significant increase in positivity rate was observed for cytomegalovirus (4.2% vs. 9.9%, OR = 2.53, *p = 0.010*), EAggEC (35.7% vs. 45.3%, OR = 1.49, *p = 0.014*), *D. fragilis* (2.0% vs. 7.8%, OR = 3.97, *p = 0.004*) and *Strongyloides* spp. (0.8% vs. 3.6%, OR = 4,42, *p = 0.048)*. Also, an increase in the positivity rate was observed for adenovirus 40/41 (5.8% vs. 7.3%, OR = 1.28, *p = 0.444*), norovirus GI/GII (11.2% vs. 15.9%, OR = 1.5, *p = 0.089*), *Shigella* spp. (8.7% vs. 13.0%, OR = 1.57, *p = 0.092*) and *Cryptosporidium * spp. (11.6% vs. 14.7%, OR = 1.31, *p = 0.261*) post-vaccine introduction but this did not reach statistical significance (Table 2). A significant decrease in test positivity rate was observed for sapovirus (7.8% vs. 4.0%, OR = 0.49, *p = 0.030*).

**Table 2:** Univariate and multivariate logistic regression comparing enteropathogen infection in the pre- vs post-rotavirus vaccine introduction periods (n = 718)

Pathogen Type	Pathogen	Total (n = 718)	Prevalence				Univariate Logistic Regression		Multivariable^#^ Logistic Regression	
			Pre-vaccine (n = 241)		Post-vaccine (n = 477)					
		n (%)	# + ve cases	Proportion (95% CI)	# + ve cases	Proportion (95% CI)	OR (95% CI)	p value	OR (95% CI)	p value
Virus	Adenovirus 40/41	49 (6.8)	14	5.8 (3.2–9.6)	35	7.3 (45.2–10.1)	1.28 (0.68, 2.44)	0.444	1.16 (0.60, 2.24)	0.667
	Any Adenovirus	104 (14.5)	28	11.6 (7.9–16.4)	76	15.9 (12.8–19.5)	1.44 (0.91, 2.29)	0.122	1.33 (0.82, 2.13)	0.247
	Rotavirus group A	178 (24.8)	66	27.4 (21.9–33.5)	112	23.5 (19.8–27.6)	0.81 (0.57, 1.16)	0.253	0.97 (0.66, 1.42)	0.879
	Norovirus GI/GII	103 (14.4)	27	11.2 (7.5–15.9)	76	15.9 (12.8–19.5)	1.50 (0.94, 2.40)	0.089	1.53 (0.94, 2.49)	0.086
	Astrovirus	34 (4.7)	12	5.0 (2.6–8.5)	22	4.6 (2.9–7.0)	0.81 (0.57, 1.16)	0.253	0.97 (0.66, 1.42)	0.879
	Sapovirus	38 (5.3)	19	7.8 (4.8–12.0)	19	4.0 (2.4–6.2)	**0.49 (0.25, 0.93)**	**0.030**	0.64 (0.32, 1.28)	0.205
	Enterovirus	193 (26.9)	65	27.0 (21.5–33.0)	128	26.8 (23.0–31.1)	0.99 (0.70, 1.41)	0.969	0.97 (0.67, 1.39)	0.851
	Parechovirus	119 (16.6)	47	19.5 (14.7–25.1)	72	15.1 (12.0–18.6)	0.73 (0.49, 1.10)	0.135	0.73 (0.48, 1.12)	0.158
	Hepatitis A virus	11 (1.5)	1	0.4 (0.0–2.3)	10	2.0 (1.0–3.8)	5.14 (0.65, 40.38)	0.120	4.00 (0.50, 31.77)	0.190
	Hepatitis E virus	1 (0.14)	0	0 (0–1.5)	1	0.2 (0.0–1.2)	NA		NA	
	Cytomegalovirus	57 (7.9)	10	4.2 (2.0–7.5)	47	9.9 (7.3–12.9)	**2.53 (1.25, 5.09)**	**0.010**	**2.81 (1.36, 5.81)**	**0.005**
Bacteria	Enteropathogenic *E. coli*	217 (30.2)	68	28.2 (22.6–34.4)	149	31.2 (27.1–35.6)	1.16 (0.82, 1.63)	0.405	1.24 (0.87, 1.76)	0.240
	Enteroaggregative *E. coli*	302 (42.1)	86	35.7 (29.6–42.1)	216	45.3 (40.8–50.0)	**1.49 (1.08, 2.05)**	**0.014**	**1.58 (1.13, 2.20)**	**0.007**
	Verotoxogenic *E. coli*	4 (0.6)	0	0 (0–1.5)	4	0.8 (0.2–2.1)	NA	0.176	NA	
	*Shigella* spp.	83 (11.6)	21	8.7 (5.5–13.0)	62	13.0 (10.2–16.4)	1.57 (0.93, 2.64)	0.092	1.49 (0.87, 2.54)	0.144
	*C. perfringens/difficile*	33 (4.6)	8	3.3 (1.4–6.4)	25	5.2 (3.4–7.6)	1.61 (0.72, 3.63)	0.250	1.51 (0.66, 3.47)	0.334
	*Salmonella * spp.	15 (2.1)	5	2.0 (0.7–4.8)	10	2.1 (1.0–3.8)	1.01 (0.34, 2.99)	0.985	1.10 (0.35, 3.42)	0.877
	*C. jejuni/coli*	56 (7.8)	17	7.1 (4.2–11.1)	39	8.2 (5.9–11.0)	1.17 (0.65, 2.12)	0.597	1.13 (0.61, 2.07)	0.706
	*V. cholerae*	11 (1.53)	0	0 (0–1.5)	11	2.3 (1.2–4.1)	NA		NA	
	*Aeromonas * spp.	7 (1.0)	3	1.3 (0.3–3.6)	4	0.8 (0.2–2.1)	0.67 (0.15, 3.02)	0.603	0.74 (0.15, 3.56)	0.707
	*Y. enterocolitica*	2 (0.3)	0	0 (0–1.5)	2	0.3 (0.0–1.5)	NA		NA	
Protozoa	*Cryptosporidium * spp.	98 (13.7)	28	11.6 (7.9–16.4)	70	14.7 (11.6–18.2)	1.31 (0.82, 2.09)	0.261	1.36 (0.83, 2.20)	0.219
	*D.fragilis*	42 (5.9)	5	2.0 (0.7–4.8)	37	7.8 (5.5–10.5)	**3.97 (1.54, 10.23)**	**0.004**	**3.90 (1.49, 10.21)**	**0.006**
	*G. lambia*	81 (11.3)	27	11.2 (7.5–15.9)	54	11.3 (8.6–14.5)	1.01 (0.62, 1.65)	0.963	0.96 (0.57, 1.61)	0.881
	*C. belli*	1 (0.14)	0	0 (0–1.5)	1	0.4 (0.0–1.2)	NA		NA	
	*C. cayetanensis*	2 (0.3)	2	0.8 (0.1–3.0)	0	0.0 (0.0–0.7)	NA	0.110	NA	
Helminth	*Strongyloides* spp.	19 (2.7)	2	0.8 (0.1–3.0)	17	3.6 (2.1–5.7)	**4.42 (1.01, 19.27)**	**0.048**	**4.60 (1.03, 20.55)**	**0.046**

^#^ All models are adjusted for age, vesikari score, fatality, length of stay (LOS), HIV status, and gender; due to high coinfection rates, enteropathogens are modelled separately; the bold emphasis of some of the* p* -values and OR (95% CI) indicate those statistically significant

In multivariable models adjusting for age, Vesikari score, fatality, gender, LOS, and HIV-status, cytomegalovirus (OR = 2.81, 95% CI 1.36, 5.81), EAggEC (OR = 1.58, 95% CI 1.13, 2.20), *D. fragilis* (OR = 3.90, 95% CI 1.49, 10.21), and *Strongyloides* spp. (OR = 4.60, 95% CI 1.03, 20.55) were all detected among significantly more patients in the post-vaccine than in the pre-vaccine period. Sapovirus no longer demonstrated a significant difference between the two time periods (Table 2).

No differences were observed for RVA pre-post-RVA vaccination (27.4% vs. 23.5%, *p = 0.253*). Under the multivariable models the risk of RVA positive status did not change after adjusting for age and receipt of recent RVA vaccination (data not shown). The absence of reduction in RVA prevalence and absence of association with vaccine status remained even when we restricted our analyses to children < 5 years. No significant changes were observed for the other remaining pathogens.

### Enteropathogen co-infections

Only 53 children (7.4%) had not even a single enteropathogen detected. Among the rest (n = 665), 148 (22.3%) were mono-infected, while 323 (48.6%) had ≥ 3 pathogens detected. Including all children, 72% (n = 517) had ≥ 2 enteropathogens detected. Five or more enteropathogens were detected in 84 children (11.7%). A greater number of different enteropathogens were detected per child in the post-vaccine compared to pre-vaccine period (mean: 2.7 vs. 2.3 respectively, *p = 0.0025*).

### Characteristics of rotavirus positive samples

**Fig. 3:**
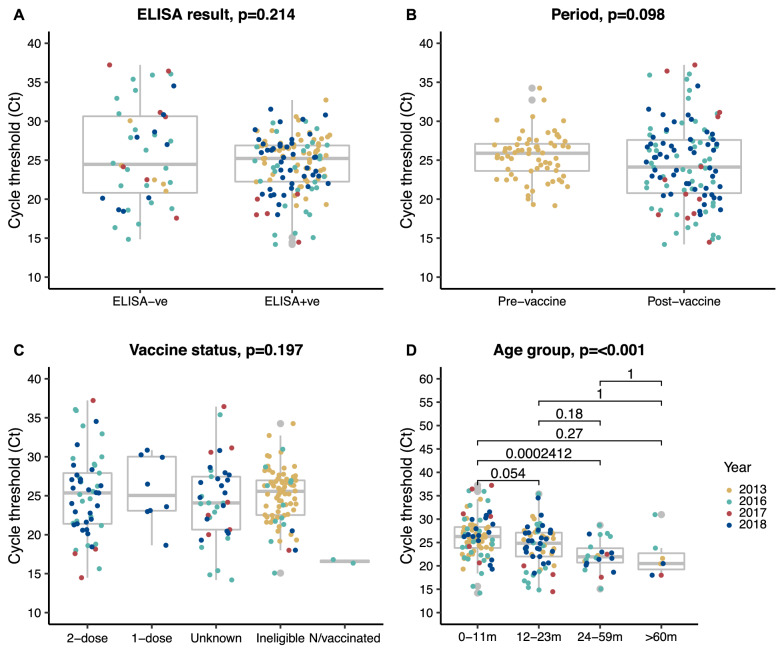
Boxplots examining the relationship between the observed RVA detection cycle threshold (Ct) from TAC assay and (**a**) previous enzyme-linked immunosorbent assay (ELISA) result on the same samples, (**b**) period of sample collection, (**c**) rotavirus vaccination status, and (**d**) age group in stool samples from 718 children at KCH.* P*-values are derived from an independent t-test, one-way ANOVA, and where applicable Kruskal Wallis was used

The distribution of RVA TAC assay Ct values for positive samples stratified by various potential influencing factors is shown in Fig. 3. The median Ct value was statistically similar between (a) RVA EIA test positive and negative samples (*p = 0.214*; Fig. 3A), (b) TAC positive samples pre- and post-vaccine introduction (Fig. 3B; *p = 0.098*), (c) across individuals who had received various numbers of rotavirus vaccine doses including the unvaccinated group (Fig. 3C; p = 0.197). However we noted that the median Ct values among older children were significantly lower than those among younger children, regardless of the sampling year (Fig. 3D; p < 0.001).

## Discussion

Our study shows very high positivity rate of enteropathogens among hospitalized children with diarrhea, extensive co-infections, and differences in pathogen positivity before and after introduction of rotavirus vaccination in this rural coastal setting of Kenya. Further, despite introduction of childhood rotavirus vaccination, RVA was the fourth most detected and we did not observe a significant difference in RVA positivity rate between pre- and post-vaccination introduction periods following TAC analysis. There have been few studies focused on illuminating on changes in multiple enteropathogen presentation post-rotavirus vaccination [26–31]. Our findings here align with previous research in low-middle income countries (LMIC) settings, such as Global Enteric Multicenter Study (GEMS) [25] and Malnutrition and Enteric Disease Study (MAL-ED) study [32], which also found high rates of enteropathogen co-infections. The changes in pathogen positivity rate pre-post rotavirus vaccination and increase in co-infection rate suggests a changing landscape of diarrhea etiology and epidemiology in Kenya. We hypothesize that the demographics of hospitalized children with severe diarrhea after rotavirus vaccination is changing to somewhat older children and those with additional risk factors such as malnutrition and HIV exposure.

The top nine enteropathogens detected in this study (in descending order) were EAggEC, EPEC, enterovirus, RVA, parechovirus, norovirus GI/GII, *Cryptosporidium* spp., *Shigella* spp. and *Giardia lambia*. A study of bacterial diarrheal infections in < 5 year-olds in Western Kenya revealed that *E. coli* and *Shigella* species were the most common bacterial infections [33]. Our previous study examining 5 viral enteropathogens among children < 5 years at KCH found RVA, and adenovirus as the commonest enteric viruses [14]. In the GEMS study which included children < 5 years-old and community controls, the top nine enteropathogens associated with diarrheal illness were RVA, enterotoxigenic *E. coli*, *Cryptosporidium* spp., *H. pylori*, norovirus GII, *Aeromonas* spp., adenovirus 40/41, EPEC, *Shigella* spp., *C. difficile*, and sapovirus [34]. A post-vaccine introduction study of hospitalized children aged < 5 years in Tanzania found RVA, ETEC, *Shigella* spp., *Cryptosporidium* spp. and astrovirus as the leading causes of diarrhea [26]. Although all the above studies took place in different geographic regions and/or used different methods of pathogen detection from the current study, there is still considerable consistency in the major enteropathogens observed.

While the enteropathogens detected in our study could cause diarrhea, several of them have been associated with asymptomatic shedding or non-diarrheic syndromes [35]. EAggEC was the most common enteropathogen detected here but several previous studies e.g., one in Malawi found EAggEC frequently in both cases (51.8%) and controls (47.8%) [28]. Generally, the role of EAggEC in acute diarrhea in sub-Saharan Africa settings remains uncertain but prolonged carriage has been associated with child growth faltering [36]. EPEC, the second most prevalent pathogen we detected here has been linked with diarrhea, for example in a Malawi study, where it was found in 18.0% of cases versus 8.3% of controls [28]. Enterovirus, the third top enteropathogen we detected has been previously associated with a wide spectrum of clinically distinct syndromes including respiratory and neurological illnesses [37]. Thus, some of the detections here could be long-term carriage unassociated with the current diarrhea episode. To better understand changes in enteropathogen epidemiology pre-post-rotavirus vaccine introduction, repeated measures studies on the same individuals including community controls are necessary [25, 32]. Such studies will also be important in understanding the longer-term impact of vaccination, carriage, and infection on children in LMIC settings.

RVA positivity rate in this study post vaccine introduction was 23.5%. RVA has been reported in several studies in sub-Sarahan African settings be the leading cause severe acute gastroenteritis (AGE) even post-vaccine introduction [26, 28]. In Tanzania, RVA was found to be a leading diarrhea hospitalization (attributable fraction (AF), 25.8%) [26] and in Malawi (AF, 34.5%) [28]. Nonetheless, the Global Rotavirus Surveillance Network showed that RVA prevalence decreased in < 5-year-olds between 2008 and 2016 following national introduction of the RVA vaccine across 82 countries, although to a lesser extent among low-income countries [24]. In Kenya, using controlled interrupted time series analysis, a > 50% decline in RVA cases were observed in two sites following Rotarix® introduction [38]. One of these sites was KCH. Further in a previous analysis, using RT-PCR, we also observed a reduction in RVA among children < 5-year-olds (23.3% vs. 13.8%) [14]. The current study found no reduction in RVA prevalence among KCH children; however, there were some key differences from the previous studies. First, to detect RVA infection, we used TAC, which is considerably more sensitive than the conventional RT-PCR [39] or EIA used in other studies [14, 40]. Second, we included children up to 13 years of age, whereas the other studies included only < 5-year-olds. Third, previous Kenyan studies, samples were collected between 2014 and 2017 [38] and 2003, 2013, 2016, and 2019 [14], which does not overlap entirely with the current study. The inclusion of older children and the use of TAC provide evidence of the overall prevalence of RVA in children, as opposed to only the vaccine eligible children [41].

Apart from the methodological differences with previous studies, the absence of a significant decline of RVA positivity post-vaccination observed here could be further due to local circulation of distinct RVA genotypes for which Rotarix ^®^ has limited efficacy. We have previously reported a temporal increase in prevalence of Rotarix ^®^ vaccine heterotypic genotypes in Kenya post-vaccination (e.g. G2P[4] and G3P[8]) [44]. It has also been reported elsewhere that RVA prevalence increased with age following introduction of vaccines [45], which might have occurred in this study that included children up to 13-years of age. Indeed overall, children from the post-rotavirus vaccine introduction period were older compared to pre-vaccine.

Studies have shown that RVA load in stool samples during an episode, as determined by RT-PCR Ct values, is significantly correlated with disease severity [46, 47]. It was found that children with more severe diarrhea excreted more virus, as evidenced by lower RT-PCR Ct values. We found that older children had significantly lower median Ct values compared with younger children. There may be a link between RVA being more severe in older children and either the non-vaccination status or the waning immune response after vaccination [48, 49]. Similar Ct values were seen in samples that were TAC positive but EIA negative, as well as in samples that were pre-vaccine versus post-vaccine. It is necessary to investigate why some TAC positives were missed by EIA to determine if there is a sensitivity issue with available EIA kits post-vaccine introduction.

The reanalysis of GEMS case-control study using TAC found *Shigella* spp./EIEC to be the topmost attributable pathogen for moderate-to-severe diarrhea [25]. Here, we observed an increase in prevalence of *Shigella* spp./EIEC pre-post rotavirus vaccine introduction although this was not statistically significant; 8.7% (95% CI 5.5–13.0%) versus 13.0 (95% CI 10.2–16.4%). The prevalence observed in this study was close to that observed in a study in neighboring Tanzania post-vaccine introduction study that found a *Shigella* spp./EIEC prevalence of 14.5% (95% CI 10.2–22.8%) [26].

Norovirus has been increasingly reported as a most important cause of sporadic and epidemic AGE post-rotavirus vaccine introduction [30, 50]. Although norovirus GI/GII was sixth as the most frequently detected enteropathogen in our study, its contribution to diarrhea etiology is likely to be significant given the suspected carriage of some of the top detected enteropathogens. Congruent with our previous findings [14], we observed its trend towards increased positivity rate pre-post-rotavirus vaccination introduction (11.2% vs. 15.9%) although this did not reach statistical significance.

We detected 11 *V. cholerae* positive cases, all occurring post-rotavirus vaccine introduction. This finding is consistent with several small outbreaks that were reported in Kenya during the period including in refugee camps [51]. The sources of this ongoing *V. cholerae* transmission in Kenya e.g. whether is a result of sustained cryptic transmission or repeated introductions into the country is unclear. In contrast to other studies in other low-resource settings, no samples in our study tested positive for *Vibrio parahaemolyticus* or *Entamoeba histolytica* [26, 32]. This was possibly due to different epidemiology for these pathogens in our setting or failure of the molecular assay we used due to primer/probe sequence mismatch [52].

This study had some limitations. Samples were collected more than two years prior to analysis, which could compromise their integrity [53]. The study setting might have experienced a further change in enteropathogen burden since 2018 in part due to the coronavirus disease 2019 (COVID-19) pandemic as has been observed elsewhere [54, 55]. However, this does not invalidate the hypotheses generated by the findings regarding enteropathogen dynamics, but rather suggests that continuous surveillance is critical for adapting and maximizing diarrheal illness prevention. We did not analyze healthy controls to adjust for the background prevalence of the detected enteropathogens in this population. Of eligible children, 56.9% provided samples, which limited our ability to assess the entire population of hospitalized children with diarrhea. As a result of health worker industrial action in Kenya, enrollment was interrupted five times [17]. In the pre-vaccine and post-vaccine introduction years, the total nucleic acid extraction procedure varied, but we did not expect this to have significantly impacted enteropathogen prevalence. Despite previous studies finding an association between pathogen quantities and diarrhea attribution, we did not attempt to model these associations in this study [25]. Given the unbalanced time series nature of this study it is difficult to draw conclusions about enteropathogen dynamics following rotavirus vaccine introduction. We reported pathogen positivity rate for study eligible children who provided a sample, but more robust analysis would require a population-based denominator to calculate population-wide incidence changes.

### Conclusions

We provide a snapshot of enteropathogens infection burden among children with diarrheal illness in a low-income setting of coastal Kenya pre-post rotavirus vaccine introduction. Our findings highlight the utility of multiplex pathogen detection arrays in enteropathogen infection surveillance to improve understanding on the causes of differential global rotavirus vaccine efficacy. Our study revealed that most children presenting with diarrheal illness in this setting were coinfected with multiple enteropathogens emphasizing the need for local improved available enteropathogen vaccine coverage and emphasis of non-pharmaceutical interventions such as WASH (water, sanitation, and hygiene) to reduce carriage and transmission of these enteropathogens [56]. Overall, the TAC platform provides a fast, broad, and accurate diagnostic tool for broad routine surveillance of enteropathogens and quick screening of outbreak samples to identify potential etiological pathogens [57]. This platform has potential to offer insight into the patient infection profiles in a clinical setting to support high-quality interventions and individual patient care. The clinical implications of the frequent coinfections and if this should influence patient care during and post admission requires follow-up investigation.

## Supplementary Information

**Additional file 1: Table S1.** Details of the targets on the Gastro v4.0K

## Data Availability

The data described in this manuscript can be accessed by via the link 10.7910/DVN/VBCUV3. Further information can be accessed submitting a request form to our Data Governance Committee (http://kemri-wellcome.org/about-us/#ChildVerticalTab_15).
