# Supplementary material for: Differences in epidemiology of enteropathogens in children pre- and post-rotavirus vaccine introduction in Kilifi, coastal Kenya
Source: Gut Pathog. 2022 Aug 1;14:32. doi: 10.1186/s13099-022-00506-z (PMC9340678; doi:10.1186/s13099-022-00506-z)
Supplement: Supplementary file 1 — Additional file 1: Table S1. Details of the targets on the Gastro v4.0K [file 13099_2022_506_MOESM1_ESM.docx]

**S1 Table.** Details of the targets on the Gastro v4.0K

| Pathogen Class | Pathogen/target | # of assays on Gastro v4.0K card | Gene (s) targeted | How positives were defined | Reference |
| --- | --- | --- | --- | --- | --- |
| Viruses | Adenovirus | 2 | Hexon gene | Both assays Ct<35.0 | [5] |
|  | Adenovirus 40/41 | 1 | Fiber gene | Ct <35.0 & Adenovirus +ve | [11] |
|  | Astrovirus | 2 | ORF2 & Capsid | Both assays Ct <35.0 | [5] |
|  | Sapovirus | 2 | RdRp | Either assay Ct <35.0 | [5] |
|  | Norovirus GI | 2 | ORF1/ORF2 | Either assay Ct <35.0 | [8] |
|  | Norovirus GII | 1 | ORF1/ORF2 | Ct <35.0 | [8] |
|  | Rotavirus group A | 2 | NSP3 | Both assays Ct <35.0 | [5] |
|  | Rotarix vaccine | 1 | NSP2 | Ct <35.0 and RVA +ve | [3] |
|  | Enterovirus | 2 | 5' UTR | Both assays Ct <35.0 | [2] |
|  | Hepatitis A virus | 1 | 5' UTR | Ct <35.0 | This study |
|  | Hepatitis E virus | 1 | ORF3 | Ct <35.0 | [9] |
|  | Parechovirus | 1 | 5' UTR | Ct <35.0 | This study |
|  | Cytomegalovirus | 1 | Immediate early gene | Ct <35.0 | This study |
| Bacteria | *Aeromonas hydrophilia* | 1 | aerolysin gene | Ct <35.0 | [6] |
|  | *Campylobacter coli/jejuni* | 1 | CadF gene | Ct <35.0 | [5] |
|  | *Campylobacter coli* | 1 | *ceuE gene* | Ct <35.0 & *Campylobacter spp* +ve | [1] |
|  | *Campylobacter jejuni#2* | 1 | mapA gene | Ct <35.0 & *Campylobacter spp* +ve | [1] |
|  | *Clostridium difficile* | 2 | GDH, ToxB | Both assays Ct <35.0 | [7] |
|  | *Clostridium perfringens* | 1 | α toxin | Ct <35.0 | 11 |
|  | *E. coli* EAEC | 1 | aggR gene | Ct <35.0 | This study |
|  | *E. coli* EPEC | 1 | *eae* | Ct <35.0 | [5] |
|  | *E. coli* VTEC | 2 | *vtx1* and *vtx2* | Either assays Ct <35.0 | [5] |
|  | *Salmonella spp* | 2 | *ttr* and *hilA* |  | 12 & 15 |
|  | *Shigella spp/*EIEC | 1 | *ipaH* |  | [5] |
|  | *Vibrio cholerae* | 1 | Tox R | Ct <35.0 | [5] |
|  | *Vibrio parahaemolyticus* | 1 | Tox R | Ct <35.0 | This study |
|  | *Yersinia enterocolitica* | 1 | lysP gene | Ct <35.0 | Liu et al 2 |
| Protozoa | *Cryptosporidium spp* | 2 | 18S rRNA and DNA J like gene | Ct <35.0 | [5] |
|  | *Cyclospora cayetanensis* | 1 | rRNA ITS2 | Ct <35.0 | [4] |
|  | *Cytoisospora belli* | 1 | 5.8S/ITS2 rRNA | Ct <35.0 | [10] |
|  | *Dientamoeba fragilis* | 1 | 5.8S rRNA | Ct <35.0 | 3 |
|  | *Entamoeba histolytica* | 1 | 18S rRNA | Ct <35.0 | [5] |
|  | *Giardia lambia* | 2 | 18S rRNA | Ct <35.0 | [5] |
|  |  |  |  |  |  |
| Helminth | *Strongyloides stercoralis* | 1 | 18S rRNA | Ct <35.0 | [12] |
|  |  |  |  |  |  |
| Controls | 16s Bacterial RNA | 1 | 16S rRNA | Ct <35.0 | This study |
|  | 18s Bacterial RNA | 1 | 18S rRNA | Ct <35.0 | Applied Biosystems |
|  | MS2 Bacteriophage | 1 | MS2g1 | Not applicable | [5, 8] |
| Total |  | 48 |  |  |  |

Ct stands for cycle threshold, ORF stands for open reading frame

Reference:

1. Best E. L., Powell E. J., Swift C., Grant K. A., Frost J. A. Applicability of a rapid duplex real-time PCR assay for speciation of Campylobacter jejuni and Campylobacter coli directly from culture plates. FEMS Microbiol Lett **2003**; 229(2): 237-41.

2. Clark T. W., Medina M. J., Batham S., Curran M. D., Parmar S., Nicholson K. G. Adults hospitalised with acute respiratory illness rarely have detectable bacteria in the absence of COPD or pneumonia; viral infection predominates in a large prospective UK sample. J Infect **2014**; 69(5): 507-15.

3. Gautam R., Esona M. D., Mijatovic-Rustempasic S., Ian Tam K., Gentsch J. R., Bowen M. D. Real-time RT-PCR assays to differentiate wild-type group A rotavirus strains from Rotarix(®) and RotaTeq(®) vaccine strains in stool samples. Hum Vaccin Immunother **2014**; 10(3): 767-77.

4. Lalonde L. F., Gajadhar A. A. Highly sensitive and specific PCR assay for reliable detection of Cyclospora cayetanensis oocysts. Appl Environ Microbiol **2008**; 74(14): 4354-8.

5. Liu J., Gratz J., Amour C., et al. A laboratory-developed TaqMan Array Card for simultaneous detection of 19 enteropathogens. J Clin Microbiol **2013**; 51(2): 472-80.

6. Liu Jie, Gratz Jean, Maro Athanasia, et al. Simultaneous Detection of Six Diarrhea-Causing Bacterial Pathogens with an In-House PCR-Luminex Assay. Journal of Clinical Microbiology **2012**; 50(1): 98-103.

7. McElgunn C. J., Pereira C. R., Parham N. J., et al. A low complexity rapid molecular method for detection of Clostridium difficile in stool. PLoS One **2014**; 9(1): e83808.

8. Rolfe K. J., Parmar S., Mururi D., et al. An internally controlled, one-step, real-time RT-PCR assay for norovirus detection and genogrouping. J Clin Virol **2007**; 39(4): 318-21.

9. Rolfe K. J., Curran M. D., Mangrolia N., et al. First case of genotype 4 human hepatitis E virus infection acquired in India. J Clin Virol **2010**; 48(1): 58-61.

10. Taniuchi M., Verweij J. J., Sethabutr O., et al. Multiplex polymerase chain reaction method to detect Cyclospora, Cystoisospora, and Microsporidia in stool samples. Diagn Microbiol Infect Dis **2011**; 71(4): 386-90.

11. Tiemessen Caroline T., Nel Marietha J. Detection and typing of subgroup F adenoviruses using the polymerase chain reaction. Journal of Virological Methods **1996**; 59(1): 73-82.

12. Verweij J. J., Canales M., Polman K., et al. Molecular diagnosis of Strongyloides stercoralis in faecal samples using real-time PCR. Trans R Soc Trop Med Hyg **2009**; 103(4): 342-6.
